# Health-Related Support and Control on Poor Diet in Mixed-Weight, Older Gay Married Couples

**DOI:** 10.1093/geroni/igaa057.1857

**Published:** 2020-12-16

**Authors:** Joshua Novak, Stephanie Wilson, Terry Peak, Julie Gast, Maya Miyairi-Steel

**Affiliations:** 1 Auburn University, Auburn, Alabama, United States; 2 Southern Methodist University, Dallas, Texas, United States; 3 Utah State University, Logan, Utah, United States

## Abstract

The health support and control literature in same sex couples suggests partners take turns (known as health behavior work). However, research has not accounted for mixed-weight (lighter partner (LP) and heavier partner (HP)) status in these relationships nor investigated domain-specific health behavior work (i.e., dietary habits) on outcomes. We examined an actor-partner interdependence mediation model of 224 mixed-weight, older gay married couples (mean age: 52) and controlled for education, relationship satisfaction and length, frequency of eating meals together, and income. Nutrition-specific eating encouragement and discouragement was not associated with poor dietary behaviors for either partner. More frequent couples’ reports of disagreements about nutrition was associated poorer diet for both the HP and LP (through their own higher depressive symptoms). Interestingly, the higher LP’s health support was associated with better LP’s diet (through lower depressive symptoms), and higher HP’s health control was associated with poorer LP’s diet (through lower depressive symptoms).

